# Assessing Infertility Literacy and Knowledge Gaps Among Patients with Cystic Fibrosis

**DOI:** 10.5152/tud.2023.23061

**Published:** 2023-09-01

**Authors:** Farah Rahman, Katherine Campbell, Nicholas Deebel, Armin Ghomeshi, Mohammed Zarli, Lisa Domaradzki, Maria Gabriela Tupayachi Ortiz, Ranjith Ramasamy

**Affiliations:** 1 Desai Sethi Urology Institute, Miller School of Medicine, University of Miami, Miami, FL, USA; 2 Department of Urology, Wake Forest University School of Medicine, Winston Salem, NC, USA; 3 Herbert Wertheim College of Medicine, Florida International University, Miami, FL, USA; 4 Dr. Kiran C Patel College of Osteopathic Medicine, Nova Southeastern University, Fort Lauderdale, FL, USA; 5 Department of Pulmonology, University of Miami, Miami, FL, USA

**Keywords:** Infertility, patient education, assisted reproductive technology, cystic fibrosis, azoospermia

## Abstract

**Objective:**

As patients with cystic fibrosis live longer into their reproductive years, fertility concerns are rising. We hypothesized that while patients with cystic fibrosis may be informed of the impact of their disease on their reproductive potential, they remain unaware of the promising role of assisted reproductive technology in helping them conceive biological children.

**Methods:**

We distributed a voluntary and anonymous survey to cystic fibrosis patients and organizations to assess patient understanding of cystic fibrosis-related infertility. The survey questions aimed to capture demographic information, their reproductive education regarding cystic fibrosis, and their preferences for future fertility.

**Results:**

Forty respondents completed the survey (median age of 36 ± 14 years). The median age reported for learning about cystic fibrosis-associated infertility was 18 years. Respondents preferred that reproductive and infertility education be provided early; 43% reported the optimal age of education was younger than 18 years while 50% reported between 18 and 24 years. Of the respondents trying to conceive, 43% of patients have been trying to conceive for 1-3 years qualifying for infertility. Yet, the majority of those patients (69%) have not been offered a semen analysis and 90% have not had previous fertility treatments.

**Conclusion:**

Our findings highlight that cystic fibrosis patients are knowledgeable about cystic fibrosis-related impacts on their fertility, with high-rated self-confidence. A fraction of patients still desire to conceive but have not been provided with assisted reproductive services. We recommend the establishment of active partnerships between cystic fibrosis care teams and fertility specialists to maximize their chances of conception.

**Main Points**

- Our surveyed cystic fibrosis (CF) patients demonstrated a strong understanding of CF-related infertility pathophysiology, with high-rated self-confidence in knowledge.
- Cystic fibrosis patients report a preference for early initiation of physician-led discussions on CF-related infertility and reproductive issues during their adolescence.
- Despite having a strong understanding of CF-related infertility, a subset of patients still express a desire for biological conception.
- Cystic fibrosis patients who maintain a desire for biological conception would find it beneficial to engage in discussions with reproductive specialists, who can explore the potential of assisted reproductive techniques as a means to assist them in achieving their goal of conceiving.

## Introduction

Cystic fibrosis (CF) is an autosomal recessive genetic condition caused by mutations in the cystic fibrosis transmembrane conductance regulator (CFTR) gene. Mutations in the CFTR gene have been demonstrated to reduce the fertility of men and women with CF. Infertility in women with CF is less prevalent than with their male CF counterparts with 35%-50% of women with CF reporting subfertility or infertility.^1^ Unlike in men, CFTR mutations in women do not alter reproductive anatomy, rather, the CFTR mutation primarily affects the function of the epithelial cells which line the reproductive tract resulting in physicochemical abnormalities. Similar to the alteration in secretion viscosity in the lungs of CF patients, functional changes in the lining of the uterus and fallopian tubes result in overly thick mucus which can impair the ability of sperm to penetrate and successfully fertilize the egg. Despite this, nearly 50% of women with CF are able to naturally conceive a biological child.^2^

Existing literature has demonstrated that over 98% of men with CF and CFTR mutations will experience infertility secondary to defects in mesonephric duct derivatives such as a congenital bilateral absence of the vas deferens (CBAVD). They may also demonstrate atretic seminal vesicles and epididymis.^3^ All of these contribute to obstructive azoospermia which is characterized by low semen volume (0.5-0.7 mL), an acidic pH due to low concentration of fructose and α-glucosidase, and no sperm.^4,5^ There are notable cases of infertility in men with CFTR mutation that demonstrate nonobstructive infertility. In such cases, researchers analyzed the involvement of CFTR in other processes such as spermatogenesis and sperm capacitation which both rely on modification fluid and electrolyte exchange within the epididymis.^2^

Despite pathologic alterations in the structure and function of the reproductive system in women and men with CF, assisted reproductive technology (ART) such as intrauterine insemination (IUI) and in vitro fertilization (IVF) remain options for patients to have biological children. Though the use of ART techniques in this patient population is relatively new, outcomes have been good with high rates of live births.^6,7^ In women with CF who are not able to conceive naturally, IVF and IUI are potential options which allow women with CF to overcome the hurdle of overly thickened mucus by fertilization in a dish or delivery of sperm directly into the uterus. For men with CF, surgical sperm extraction allows for their sperm to be used for IVF. An added positive of IVF is genetic testing prior to implantation. However, because surgical sperm retrieval methods and subsequent IVF or IUI are costly and invasive, it is imperative that both male and female CF patients be educated about their fertility limitations and potential fertility treatment options in a sustained and time-sensitive matter.

With an increasing median age of survival (40-50 years) since the introduction of CFTR modulators, there is a progressive change in survival allowing adults with CF to consider the new prospective of parenthood.^8^ Cystic fibrosis patients and physicians must know the most appropriate time to discuss reproductive needs in the setting of reduced fertility/infertility and the potential role of ART. While varied genitourinary and sexual health concerns exist among CF patients, this project assessed the gaps in infertility knowledge among men and women with CF, with the goal to understand patient preferences on how to combat these challenges and develop streamlined solutions to better the network of CF healthcare team including fertility specialists. We proposed that individuals with CF who perceived a deficiency in their reproductive education would express a significant interest in enhancing this area of teaching in their disease framework.

## Material and Methods

This study was reviewed and approved as exempt status by the Institutional Review Board (IRB) exemption from the University of Miami as non-human research (IRB No. 20221032). We distributed a voluntary and anonymous survey using Qualtrics to CF patients and advocacy organizations to assess patient understanding of fertility and reproductive processes within CF. Prior to accessing the survey, participants were required to read through and sign an informed consent form on the primary page of the Qualtrics webpage. The survey consisted of 21 questions and was meant to capture demographics, reproductive knowledge (pathophysiology of infertility in CF and options for conception), preferences on reproductive education, and current conception practices (full questionnaire provided in Appendix A). Questions regarding preferences were scored on the Likert scale, confidence regarding reproductive knowledge was assessed using percentages, and free text response was used to assess personal experiences.

### Statistical Analysis

Statistical analysis was performed on SAS Studio Software, version 5.2. Descriptive statistics were calculated for respondents’ demographics, preferences, and attitudes and presented as percentanges. Continuous variables were presented either as mean ± standard deviation or medians and interquartile ranges in accordance with the data distribution. 

## Results

### Demographics

A total of 41 participants started the survey (median age of 36 ± 14 years, with 1 respondent withdrawing from the survey before completion. An overwhelming majority of survey takers identified as Caucasian (80.5%) and non-Hispanic (69%). The highest level of education varied variably between high school level education to doctorate level, with the most common being a bachelor’s degree (29.3%). Insurance coverage included a wide range of options, with the most common options being employer-based plan, Medicare, and private plans. Most participants in the study were either married (45.0%) or single (37.5%), while smaller proportions of individuals reported being divorced (12.5%) or widowed (5.0%). The larger part of participants (57.5%) reported not having children, while smaller proportions indicated being a parent to a child that is not biologically theirs (7.5%), having biological children (30.0%), or having adopted a child (5.0%). This is due to both men and women participating in our survey. Survey responder demographics are outlined in Table 1.

### Cystic Fibrosis-Related Reproductive Education

When asked to rate their level of confidence in their knowledge of how CF impacts fertility, on a percentage scale ranging from 0 to 100%, participants averaged 79% ± 26.9%.

Seventy-five percent of patients correctly identified the vas deferens as the site of anomaly, leading to infertility in men with CF.

### Preferences on Fertility and Reproductive Health Education

The median age that participants first became aware of fertility issues associated with CF was 18 years (quartile range 11 years). The breakdown of where the participant first learned about infertility varied between the healthcare team (33%), media (18%) and parents or family members (13%). One participant reported learned about it while taking the survey. Half of the participants (50%) said 18-24 years old is the best time to counsel CF patients on reproductive health and infertility, while 43% said they would prefer this to be done at an age younger than 18 years old. Upwards of three-fourths of survey takers (77%) regarded the inclusion of sexual and reproductive health knowledge within the CF treatment plan as “extremely important” or “very important,” the remaining 23% identified with “moderately” or “slightly” important.

### Fertility Data

When asked about future conception preferences, 53% of participants said “No, they do not want to conceive.” Of the patients who are currently trying to conceive, 43% have been trying for 1-3 years. However, an overwhelming majority of these individuals (69%) struggling with fertility and conception have yet to be offered a semen analysis by their provider. Most importantly, 90% of all our respondents report not having undergone any fertility treatments previously.

## Discussion

In addition to the well-known impacts of CF on the lungs and gastrointestinal system, infertility remains a common phenotype of patients with CFTR mutations. In men, a hallmark of the disease is CBAVD resulting in obstructive azoospermia and male infertility. While this is a well-established fact and well known to medical providers, we aimed to identify if this information is properly conveyed to patients with CF and if so at what age. Moreover, we aimed to understand if the subject of infertility and treatment options has been revisited in adulthood, if desired. The identification of gaps or latency in fertility education, treatment, and procedure availability creates a unique opportunity to develop streamlined communication between patients, partners, CF care teams, and fertility specialists.

To assess patient preferences and knowledge of CF-specific reproductive literacy, we conducted one of the largest cross-sectional assessments to date including 41 patients with CF. While the median age that our cohort learned about infertility secondary to CF was 18 years old, 1 patient reported learning about the impact of CF on fertility through participation in this study. When queried about the “ideal” age for teaching patients about the role of CFTR mutation on fertility, the majority (93%) of respondents noted it should be before age 24. This is consistent with the work of Hailey et al^9^ who surveyed 20 adults with CF about pursuing parenthood and reported that the majority of patients stated the appropriate window for education would be in the “mid to late teenage” years.

Of the small fraction of our patients who are currently attempting to conceive (20%), nearly half have been attempting pregnancy for 1-3 years, qualifying themselves as a couple experiencing infertility.^10^ Yet, only 31% of patients interested in achieving paternity had been offered a semen analysis. Previous work showed that male CF patients desired to be offered an initial semen analysis in late adolescence.^11,12^ And only 10% have undergone previous fertility treatments. The lack of fertility work-up and consideration for fertility treatment may reflect the archaic notion that patients with chronic diseases such as CF are not interested in conception or parenthood.^13^

The findings in our study are consistent with other work which has shown that patients with CF believe that the inclusion of reproductive healthcare into their treatment plan is important.^14-16^ The median age of infertility education was 18 years; however, the current median age of survey respondents is 36 years (quartile range of 14). Since participants voluntarily chose to participate in our survey years after they report learning about the fertility implications of CF and continue to report that reproductive education is of high importance, it appears that sexual and reproductive health concerns are truly an important part of improving the quality of life of patients. While we do not know how important they regarded their fertility at the time of education, we do know that at this point in time a fraction of our survey respondents will attempt to conceive. This underscores the need for sustained and continued communication with CF patients through different phases of life with multidisciplinary providers so that reproductive and fertility education can be tailored to that phase of life.

A novel finding of our study was that patients surveyed were knowledgeable and self-confident about CF-related reproductive abnormalities. We found that 75% of patients correctly identified the vas deferens as the site of anomaly leading to infertility in men with CF. Additionally, patients reported a high level of self-confidence with 79% feeling confident in their knowledge of the limitations on their reproductive abilities. This finding suggests that despite some patients learning of their fertility potential in a delayed fashion, patients are adequately educated about the details of their condition in regard to fertility. Despite this knowledge, we found that 20% of survey respondents were actively trying to conceive or were interested in conceiving in the future. Underlying the concept, that while patients with CF understand their lowered reproductive potential, a portion of CF patients continue to desire conception with their partners. Offering the opportunity and need to educate this subsect of CF patients about assisted reproductive techniques would make reproduction possible.

Our study is not without limitations. This was a single-center, cross-sectional study. Due to the nature of surveying both sexes, the experiences may not be generalizable to the greater male CF patient population. Therefore, multi-institutional studies are needed in the future to confirm the results of this study. Another important limitation of this study is the possibility of recall bias with retrospective questions about fertility education. Finally, we did not ask about urologic conditions or sexual health concerns besides fertility in this population. Given the diverse impacts of CF mutations on the genitourinary system, we may have missed an opportunity to learn about educational practices and desires from patients who have sexual and reproductive health concerns aside from fertility. Despite these limitations, this survey represents a large study of the fertility and educational desires of patients. With CF patients living longer and healthier lives with CFTR modulators, there is a growing need for continued discussion about infertility diagnosis and treatment options for CF patients who desire to conceive via assisted reproductive techniques. This work is an important step toward providing optimal, patient-centric care and the development of care that matches the wishes of patients.

## Figures and Tables

**Table 1.** Study Demographics of Survey Participants

		Total (n = 41)
Age (mean ± STD ) [years]^*^		36 ± 14.2
Race	White	33 (80.5%)
	Black	3 (7.3%)
	Other	5 (12.2%)
Ethnicity	Hispanic/Latino	13 (31.7%)
	Non-Hispanic/non-Latino	28 (68.3%)
Highest educational level	High school graduate	5 (12.2%)
	Some college	9 (22%)
	Associate’s degree	5 (12.2%)
	Bachelor’s degree	12 (29.3%)
	Master’s degree or professional degree	8 (19.5%)
	Doctorate degree	2 (4.8%)
Insurance coverage	Private plan	9 (22%)
	Employer plan	14 (34.2%)
	Medicare	10 (24.4%)
	Medigap	1 (2.4%)
	Medicaid	6 (14.6%)
	Military	1 (2.4%)
Relationship status	Single	15 (37.5%)
	Married	18 (45.0%)
	Divorced	5 (12.5%)
	Widowed	2 (5.0%)
Parental status	No children	23 (57.5%)
	I parent a child that is not mine	3 (7.5%)
	I have biological children	12 (30.0%)
	I have adopted a child	2 (5.0%)

## References

[1] Sueblinvong V Whittaker LA . Fertility and pregnancy: common concerns of the aging cystic fibrosis population. Clin Chest Med. 2007;28(2):433 443. (10.1016/j.ccm.2007.02.009) 17467558

[2] Ahmad A Ahmed A Patrizio P . Cystic fibrosis and fertility. Curr Opin Obstet Gynecol. 2013;25(3):167 172. (10.1097/GCO.0b013e32835f1745) 23429570

[3] Yoon JC Casella JL Litvin M Dobs AS . Male reproductive health in cystic fibrosis. J Cyst Fibros. 2019;18(suppl 2):S105 S110. (10.1016/j.jcf.2019.08.007) 31679721

[4] Chen H Ruan YC Xu WM Chen J Chan HC . Regulation of male fertility by CFTR and implications in male infertility. Hum Reprod Update. 2012;18(6):703 713. (10.1093/humupd/dms027) 22709980

[5] de Souza DAS Faucz FR Pereira-Ferrari L Sotomaior VS Raskin S . Congenital bilateral absence of the vas deferens as an atypical form of cystic fibrosis: reproductive implications and genetic counseling. Andrology. 2018;6(1):127 135. (10.1111/andr.12450) 29216686 PMC5745269

[6] Popli K Bourke S Stewart J . Fertility issues in men with cystic fibrosis: survey of knowledge and opinion of patients. Fertil Steril. 2009;91(4 suppl):1297 1298. (10.1016/j.fertnstert.2008.01.048) 18851849

[7] Persily JB Vijay V Najari BB . How do we counsel men with obstructive azoospermia due to CF mutations?-a review of treatment options and outcomes. Transl Androl Urol. 2021;10(3):1467 1478. (10.21037/tau-19-681) 33850781 PMC8039579

[8] Bell SC Mall MA Gutierrez H , et al. The future of cystic fibrosis care: a global perspective. Lancet Respir Med. 2020;8(1):65 124. (10.1016/S2213-2600(19)30337-6) 31570318 PMC8862661

[9] Hailey CE Tan JW Dellon EP Park EM . Pursuing parenthood with cystic fibrosis: reproductive health and parenting concerns in individuals with cystic fibrosis. Pediatr Pulmonol. 2019;54(8):1225 1233. (10.1002/ppul.24344) 31066212 PMC6642021

[10] Schlegel PN Sigman M Collura B , et al. Diagnosis and treatment of infertility in men: AUA/ASRM guideline part II. Fertil Steril. 2021;115(1):62 69. (10.1016/j.fertnstert.2020.11.016) 33309061

[11] Tsang A Moriarty C Towns S . Contraception, communication and counseling for sexuality and reproductive health in adolescents and young adults with CF. Paediatr Respir Rev. 2010;11(2):84 89. (10.1016/j.prrv.2010.01.002) 20416543

[12] Fair A Griffiths K Osman LM . Attitudes to fertility issues among adults with cystic fibrosis in Scotland. The collaborative group of Scottish adult CF centres. Thorax. 2000;55(8):672 677. (10.1136/thorax.55.8.672) 10899244 PMC1745836

[13] Jain R Kazmerski TM Zuckerwise LC , et al. Pregnancy in cystic fibrosis: review of the literature and expert recommendations. J Cyst Fibros. 2022;21(3):387 395. (10.1016/j.jcf.2021.07.019) 34456158

[14] Sawyer SM Farrant B Cerritelli B Wilson J . A survey of sexual and reproductive health in men with cystic fibrosis: new challenges for adolescent and adult services. Thorax. 2005;60(4):326 330. (10.1136/thx.2004.027599) 15790989 PMC1747378

[15] Hull SC Kass NE . Adults with cystic fibrosis and (in)fertility: how has the health care system responded? J Androl. 2000;21(6):809 813 11105906 PMC4819317

[16] Clarke AR Stransky OM Bernard M , et al. Exploring provider attitudes and perspectives related to men’s health in cystic fibrosis. J Cyst Fibros. 2022;21(4):652 656. (10.1016/j.jcf.2021.12.016) 34998704

